# Cognitive and Affective Perspective-Taking: Evidence for Shared and Dissociable Anatomical Substrates

**DOI:** 10.3389/fneur.2018.00491

**Published:** 2018-06-25

**Authors:** Meghan L. Healey, Murray Grossman

**Affiliations:** ^1^Penn Department of Neurology and Frontotemporal Degeneration Center, University of Pennsylvania Perelman School of Medicine, Philadelphia, PA, United States; ^2^Neuroscience Graduate Group, University of Pennsylvania Perelman School of Medicine, Philadelphia, PA, United States

**Keywords:** perspective-taking, empathy, cognitive, affective, emotion, frontotemporal dementia, neuroimaging

## Abstract

Perspective-taking refers to the ability to recognize another person's point of view. Crucial to the development of interpersonal relationships and prosocial behavior, perspective-taking is closely linked to human empathy, and like empathy, perspective-taking is commonly subdivided into cognitive and affective components. While the two components of empathy have been frequently compared, the differences between cognitive and affective perspective-taking have been under-investigated in the cognitive neuroscience literature to date. Here, we define cognitive perspective-taking as the ability to infer an agent's thoughts or beliefs, and affective perspective-taking as the ability to infer an agent's feelings or emotions. In this paper, we review data from functional imaging studies in healthy adults as well as behavioral and structural imaging studies in patients with behavioral variant frontotemporal dementia in order to determine if there are distinct neural correlates for cognitive and affective perspective-taking. Data suggest that there are both shared and non-shared cognitive and anatomic substrates. For example, while both types of perspective-taking engage regions such as the temporoparietal junction, precuneus, and temporal poles, only affective perspective-taking engages regions within the limbic system and basal ganglia. Differences are also observed in prefrontal cortex: while affective perspective-taking engages ventromedial prefrontal cortex, cognitive perspective-taking engages dorsomedial prefrontal cortex and dorsolateral prefrontal cortex (DLPFC). To corroborate these findings, we also examine if cognitive and affective perspective-taking share the same relationship with executive functions. While it is clear that affective perspective-taking requires emotional substrates that are less prominent in cognitive perspective-taking, it remains unknown to what extent executive functions (including working memory, mental set switching, and inhibitory control) may contribute to each process. Overall results indicate that cognitive perspective-taking is dependent on executive functioning (particularly mental set switching), while affective perspective-taking is less so. We conclude with a critique of the current literature, with a focus on the different outcome measures used across studies and misconceptions due to imprecise terminology, as well as recommendations for future research.

## Introduction

Perspective-taking is a complex and multifaceted sociocognitive process that enables us to recognize and appreciate another person's point of view, whether it be the same or different from our own. Previous work has shown that perspective-taking is closely related to and a key aspect of human empathy, which refers to the ability to internally simulate and adopt the mental states of others. Perhaps unsurprisingly then, both perspective-taking and empathy are critical in guiding successful social interactions, effective communication, and prosocial behavior. For example, an individual's perspective-taking capacity is known to predict the size of one's social network (1, 2), and empathy is known to predict altruistic giving, prosocial behavior, and overall life satisfaction (3–5). Despite such a fundamental role in today's society, however, there is still much to be learned about the cognitive and neural underpinnings of perspective-taking.

Perspective-taking is sometimes characterized along two dimensions: cognitive and affective. Cognitive perspective-taking may be defined as the ability to infer the thoughts or beliefs of another agent, while affective perspective-taking may be defined as the ability to infer the emotions or feelings of another agent. This distinction between cognitive and affective components raises an important question: are there dissociable anatomic substrates for cognitive and affective perspective-taking? Or, is there an independent perspective-taking module that can be applied to either emotional content or cognitive content?

**Figure 1:**
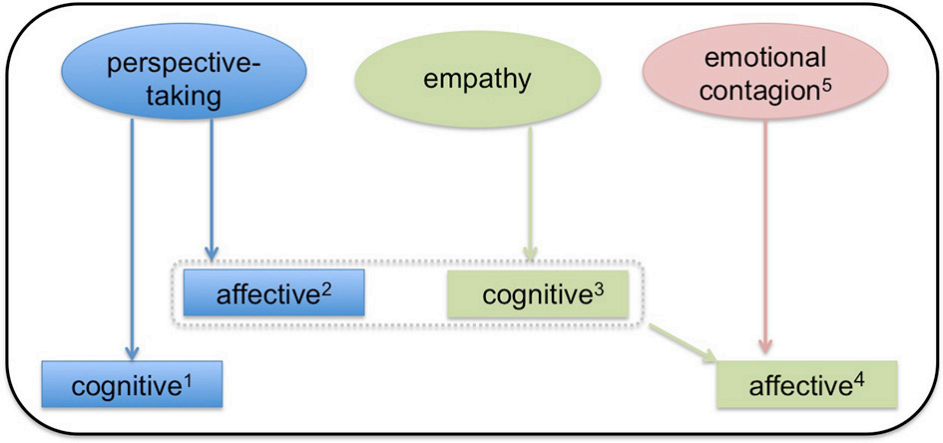
A model of the relationship between empathy and perspective-taking. In this model, both perspective-taking and empathy are subdivided into cognitive and affective components. ^1^Cognitive perspective-taking refers to the ability to make inferences about others' thoughts and beliefs. ^2^Affective perspective-taking is the ability to make inferences about others' emotions and feelings. Affective-perspective taking is thus very closely related to cognitive empathy (illustrated by the dashed box). ^3^Cognitive empathy is ability to model another agent's emotions. It may be a prerequisite to affective empathy. ^4^Affective empathy results from a combination of cognitive empathy and emotional contagion. Here, the perceiver not only models the other agent's emotion, but also adopts it (i.e., affect sharing). Affect sharing thus distinguishes affective empathy from affective perspective-taking. ^5^Emotional contagion refers to the process by which emotions in one agent trigger isomorphic emotions in another agent. Emotional contagion may occur without conscious awareness.

This line of inquiry has been considered more commonly in the context of empathy, which is frequently divided into cognitive and affective components. Note that while there is agreement that these two different types of empathy exist, the terms themselves are imprecise and a variety of alternatives are offered throughout the cognitive neuroscience literature (6). Here, in this review, we define cognitive empathy as the ability to model the emotional states of others (e.g., “I understand what you feel”). As shown in Figure 1, this definition of cognitive empathy makes it tantamount to affective perspective-taking. Other commonly used terms include affective theory of mind and mentalizing, although we stress that these terms are poorly operationalized. Next, we define affective empathy as the ability to share the emotional experience of others (i.e., “I feel what you feel”). Affective empathy may also be referred to as experience-sharing and affect-sharing, among others. Thus, while both cognitive and affective empathy depend upon perspective-taking, the difference between the two processes is based on whether or not an individual not only recognizes but also adopts the other agent's emotion. This concept of affective empathy is related to emotional contagion, which refers to the automatic and primitive process by which observation of emotions in one agent triggers isomorphic emotions in a second agent. When an agent both experiences another's emotions (i.e., emotional contagion) and models them effectively (i.e., cognitive empathy/affective perspective-taking), affective empathy results. See Figure 1 for a visual depiction of the relationship between emotional contagion and empathy.

These two types of empathy, cognitive and affective, may map onto two components of empathic processing, although there is some debate on how these components may interact. One model specifies that cognitive and affective empathy are dissociable: they operate independently and depend on unique neural substrates (6–8). An alternative model suggests that the two are part of a single system and may even operate in sequence, such that one must first recognize the other agent's emotion and identify with it, and then successfully attribute the source of the emotion to the agent and inhibit one's own perspective (9, 10). A more recent model (10) synthesizes these two positions and proposes both shared and unique neural substrates.

To date, it appears that the majority of data, from both functional imaging studies in healthy adults and lesion studies in patients, support the view that cognitive and affective empathy are largely distinct processes (8, 11). For example, Shamay-Tsoory et al. (8) found a behavioral and anatomic double dissociation between cognitive and affective empathy: patients with lesions in the ventromedial prefrontal cortex showed a selective deficit in cognitive empathy and theory of mind while patients with lesions in the inferior frontal gyrus showed selective deficits in affective empathy and emotion recognition.

While there is mostly a consensus that the two types of empathy are in part dissociable processes (6), less discussed is whether or not there are unique anatomic substrates underlying the two different kinds of perspective-taking. This question is of crucial import as perspective-taking is itself the key process upon which empathy depends. Importantly, the distinction between cognitive and affective-perspective taking here is a fine-grained one: unlike empathy, there is no element of experience or affect-sharing in perspective-taking. The primary distinction between cognitive and affective perspective-taking is rather the type of content that the perceiver is modeling. Accordingly, in this review, cognitive perspective-taking is defined as the ability to infer the *thoughts or beliefs* of another agent, while affective perspective-taking is defined as the ability to infer the *emotions or feelings* of another agent. See Figure 1 for the relationship between the types of perspective-taking and empathy.

In addition to the different types of content being modeled in cognitive vs. affective perspective-taking, there are potential differences in how cognitive and affective perspective-taking may relate to or depend upon executive functions. Previous work has demonstrated that affective perspective-taking, as one might imagine, is tightly linked to emotion perception (12, 13). Unknown, however, is what construct(s) cognitive-perspective-taking is related to. According to Miyake et al. (14), there are three postulated subdomains of executive function: mental set shifting, information updating and monitoring (i.e., working memory), and inhibitory control. Each of these executive functions, which are generally probed by different neuropsychological measures and supported by different brain regions, may play a unique role in cognitive or affective perspective-taking.

Therefore, we ask: what are the neural correlates of cognitive and affective perspective-taking? Are these processes supported by a single neural system or discrete neural systems? To answer these questions, we review neuroimaging studies from healthy adults and from individuals with focal neurodegenerative disease, namely, behavioral variant frontotemporal degeneration (bvFTD). We seek converging evidence for these anatomical findings by investigating if cognitive and affective perspective-taking demonstrate the same or different relationships with executive functions. Data showing that cognitive and affective perspective-taking have the same relationship with executive function would constitute evidence for a single-system model and data showing that cognitive and affective perspective-taking have different relationships with executive function would constitute evidence for a two-system model. Considered together, our findings will help (1) further our theoretical understanding of perspective-taking, (2) explain individual differences in healthy adults and patterns of impairment in clinical populations, and (3) offer potential targets for interventions designed to enhance perspective-taking behavior.

## Perspective-taking in healthy adults

Recently, functional imaging studies have begun to compare and contrast the neural correlates of cognitive and affective perspective-taking, exploring the question of whether or not there is a core module for perspective-taking or if the two processes are largely dissociable. Here, we review only papers that investigate these two processes within a single task. Overall, results seem to indicate that affective and cognitive perspective-taking are related to brain activity in overlapping but separable neuroanatomic networks. For example, Völlm et al. (15) scanned subjects while presenting them with cartoon stories. Following each story, the subject had to indicate which of two pictures showed the main character's next action (cognitive perspective-taking) or which of two pictures showed an action that would make the main character feel better (affective perspective-taking). Despite the difference in outcome measures across conditions, the authors demonstrated common areas of activation in medial prefrontal cortex and temporoparietal junction. However, affective perspective-taking (referred to as “empathic perspective-taking” by the authors and defined as the ability to infer other's emotional experiences) elicited additional activations in paracingulate, anterior and posterior cingulate cortices, and amygdala, while cognitive perspective-taking (referred to as “theory of mind” stimuli by the authors and defined as the ability to attribute mental states to others) elicited additional activations in lateral orbitofrontal cortex, middle frontal gyrus, and superior temporal gyrus. Complementary to these results are the results of Hynes et al. (16). Using short written scenarios, Hynes et al. (16) revealed a differential role of the orbitofrontal cortex in affective vs. cognitive-perspective taking, with the medial orbitofrontal cortex (i.e., Brodmann's areas 11 and 25) preferentially involved in affective perspective-taking. Corradi-Dell'Acqua et al. (17) and Sebastian et al. (18) also demonstrated different patterns of activity in prefrontal cortex when contrasting cognitive and affective perspective taking. For instance, Sebastian et al. (18) collected fMRI while adult subjects were presented with cartoon vignettes. Both cognitive and affective conditions elicited activity in temporoparietal junction, precuneus, and temporal poles, while only affective perspective-taking recruited medial/ventromedial prefrontal cortex (vmPFC). The authors interpret the vmPFC finding as evidence that this region, with its connections to the insula, temporal pole, and amygdala, is well-suited to integrate affective and non-affective information during theory of mind processing. This conclusion mirrors the previous lesion study findings of Shamay-Tsoory et al. (19, 20).

More recently, Bodden et al. (21) collected fMRI while 30 healthy adults completed the Yoni task, adapted from Shamay-Tsoory et al. (22). In the Yoni task, statements are written on the top of the screen about what object character “Yoni” prefers (affective) or is thinking of (cognitive) and the participant's task is to select the correct option. Results indicated that there are both shared and distinct anatomic correlates of cognitive and affective perspective taking. For example, classic theory of mind regions including the superior temporal sulcus/temporoparietal junction and parietal regions in the right hemisphere were associated with both conditions. However, the orbitofrontal cortex, inferior frontal gyrus, and basal ganglia were only associated with affective perspective taking. Schlaffke et al. (23) showed similar results using cartoon picture stories. A direct contrast of affective vs. cognitive perspective-taking associated regions within the prefrontal cortex, posterior cingulate cortex, and basal ganglia with affective perspective-taking. Cognitive relative to affective perspective-taking, on the other hand, revealed precuneus and bilateral temporal lobes. While this may seem to at odds with the results of Sebastian et al. (18), who found the precuneus and temporal pole were engaged in both conditions, the results are actually not inconsistent. Even though there was higher activation in the cognitive condition in the precuneus and temporal lobe in Schlaffke et al. (23), they also demonstrated an overlap in activation with the affective condition.

Finally, Kalbe et al. (24) took a different approach and used repetitive transcranial magnetic stimulation (TMS) to examine cognitive and affective perspective-taking and in particular, the role of the right dorsolateral prefrontal cortex (DLPFC). Healthy male subjects performed a computerized version of the Yoni task while a single train of 900 1 Hz TMS was applied to the right DLPFC to reduce cortical excitability. TMS stimulation produced a selective impairment of cognitive but not affective perspective-taking, suggesting that the neural networks supporting these processes are functionally independent.

In summary, the functional imaging literature seems to suggest affective perspective-taking may uniquely engage amygdala, basal ganglia, ventromedial prefrontal cortex, and inferior frontal gyrus. Cognitive perspective-taking, on the other hand, may uniquely engage the dorsomedial prefrontal cortex and DLPFC. Both processes may engage the temporoparietal junction and precuneus (25).

## Perspective-taking in frontotemporal degeneration

While fMRI can associate patterns of brain activity with ongoing behavior, it is a correlative technique that cannot identify which brain regions are truly *necessary* for a given task. Therefore, it is important to complement fMRI studies with converging evidence from patient studies. Here, we test the relationship between cognitive and affective perspective-taking by studying bvFTD. bvFTD is a young-onset neurodegenerative disease characterized by executive and social limitations due to progressive atrophy in frontal and temporal regions (26). Loss of empathy and perspective-taking are hallmark features of bvFTD (26) and have been demonstrated through a variety of tasks, including the Interpersonal Reactivity Index (IRI), the Multifaceted Empathy Test (MET), and the Story-based Empathy Task (SET) (15, 27–30). Generally speaking, these tasks, although varying in method and modality, show that patients with bvFTD struggle to accurately infer others' mental states (e.g., thoughts, feelings, intentions) accurately and consequently fail to share their emotions as well. Finally, bvFTD is an appropriate lesion model to study empathy because the patterns of atrophy that are characteristic of the disease include regions that are hypothesized to play an important role in empathy as well.

Since bvFTD is a rare clinical population, there are only a few reports that have contrasted cognitive and affective perspective-taking within a single study. Search terms here included “frontotemporal dementia” and “perspective-taking” or “frontotemporal dementia” and “theory of mind.” Studies were then narrowed down to those that contrasted cognitive and affective domains within the same patients. Most of this research focuses on the additional regions that must be engaged particularly when affective mental states are modeled. For example, Cerami et al. (31) administered the nonverbal SET, which was based on the earlier work of Vollm et al. (15). The task requires subjects to identify the correct ending of short comic strips that include intention attribution (i.e., cognitive perspective taking, as defined here), emotion attribution (i.e., affective perspective taking), or causal inference (a control condition). Despite its name, the SET does not actually assess empathy: at no point are subjects queried as to whether or not they *shared* the emotion of the main character. Thus, it is better described as a perspective-taking task. Results showed that patients with mild bvFTD were impaired in both intention attribution and emotion attribution, but were significantly worse at emotion attribution. Since the tasks were otherwise matched in difficulty, this within-group effect suggests there may be differences between the two processes. Structural imaging data also revealed differences between intention attribution and emotion attribution: although no unique regions were reported for intention attribution, results indicated that emotion attribution was uniquely related to gray matter density in the right amygdala, left posterior insula, and left posterior superior temporal sulcus extending into the temporoparietal junction. Interestingly, the finding that the temporoparietal junction is related to emotion but not intention attribution is inconsistent with the results from the healthy adult fMRI studies, which concluded that the temporoparietal junction is involved in both cognitive and affective perspective-taking. The precuneus, however, was observed for both types of attribution as expected. Importantly, no direct contrast between emotion and intention attribution was performed by Cerami et al. (31), so it remains possible that the difference in activation across conditions was not statistically significant. In confirmation of the healthy adult studies, the authors ultimately conclude that the aforementioned limbic and frontoinsular structures can be used to differentiate the two types of attribution or perspective-taking. Caminiti et al. (32) also found that mild bvFTD patients show an impaired ability to attribute cognitive and affective states to other agents using a similar version of the SET. They also examined how abnormal patterns of brain activity at rest may relate to performance, finding that patients with worse affective mentalizing performance showed weaker functional connectivity between medial prefrontal cortex and the attentional network, as well as reduced coherent activity within executive, sensorimotor, and fronto-limbic networks. These results are consistent with the earlier work of Cerami et al. (31).

There are also two behavioral studies contrasting cognitive and affective perspective-taking in bvFTD, the conclusions of which are well-aligned with the above imaging studies. For example, Torralva et al. (33) report differential cognitive and affective perspective-taking abilities at different stages of disease in bvFTD. Patients were classified as mild or moderate based on clinical disease rating (CDR) scores, with both groups showing impaired cognitive and affective perspective-taking on the faux pas recognition task (34). In the faux pas recognition task, patients read brief stories in which someone unintentionally commits a social faux pas (or not). When a faux pas is identified, patients are asked a question about the first character's intentionality (cognitive perspective-taking) and the second character's feelings (affective perspective-taking). The authors found that patients with mild bvFTD outperformed the moderate group in the cognitive condition, but not in the affective condition. Since affective perspective-taking deficits are present even at early stages of the disease, while cognitive perspective-taking is preserved, this suggests unique perspective-taking modules for each type of content. Furthermore, while cognitive perspective-taking was correlated with executive function (i.e., mental flexibility as assessed by the Wisconsin Card Sorting Task), affective-perspective taking was not. This suggests (1) there may be a core deficit in affective-perspective taking in bvFTD that is less likely due to executive deficits and (2) since cognitive and affective perspective-taking have different relationships with executive function, they may consist of two systems that are at least partially dissociable. Dodich et al. (35) obtained similar results, but using the SET described previously. The authors showed that mild bvFTD patients were impaired in both conditions relative to healthy controls. A vectorial analysis then showed that the patients were disproportionately impaired on the affective, but not cognitive condition, compared to the basic abilities (i.e., causal inference) condition, again suggesting a relative deficit in affective (but not cognitive) perspective-taking in bvFTD. Taken as a whole, the imaging and behavioral studies in frontotemporal degeneration support the argument that cognitive and affective perspective-taking are at least partially dissociable. This conclusion is based on several lines of evidence: (1) affective perspective-taking in bvFTD can be selectively impaired, (2) cognitive, but not affective, perspective-taking in bvFTD is associated with executive function performance, and (3) limbic and frontoinsular structures are uniquely related to affective perspective-taking.

Finally, while the purpose of this review is to highlight the similarities and differences between cognitive and affective perspective-taking in frontotemporal degeneration, it is important to note that there is a more extensive body of literature on empathy itself in this patient population. For example, the IRI is commonly used to examine human empathy (36) and is often administered in bvFTD, either to the patient him/herself or to a relative or caregiver. The IRI is a 28-item survey that probes 4 domains: perspective taking, fantasy, empathic concern, and personal distress. Rankin et al. (30) examined the IRI in a mixed sample of neurodegenerative disease patients. When patients with bvFTD were analyzed independently, they showed behavioral impairments in both cognitive and emotional aspects of empathy. Results also indicated that global empathy (total score on the IRI) was related to atrophy in the right subcallosal gyrus in the inferior frontal cortex. Eslinger et al. (27) expanded upon these results by investigating the individual subscales of the IRI. The authors reported that the perspective-taking subscale of the IRI was related to right dorsolateral prefrontal cortex, which is consistent with the TMS results of Kalbe et al. (24) mentioned earlier, as well as the temporal pole and subcortical structures including the right amygdala and left caudate nucleus. The perspective-taking score from the IRI was also correlated with executive measures of mental flexibility, consistent with the report of Torralva et al. (33). Many authors argue that the perspective-taking subscale of the IRI is a proxy for cognitive empathy, but a careful item analysis suggests that may be a combination of both cognitive (e.g., “I believe there are two sides to every question and try to look at them both”) and affective (e.g., “Before criticizing somebody, I try to imagine how I would feel if I were in their place”) items. Indeed, Davis (36) describes perspective taking as the “tendency to spontaneously adopt the psychological point of view others,” a definition which would encompass both cognitive and emotional mental states. Finally, regions related to empathic concern (e.g., “I often have tender, concerned feelings for people less fortunate than me”) included the right medial frontal cortex. Empathic concern, as the name suggests, is closely related to affective empathy. Therefore, a single instrument is able to yield both a measure of perspective-taking and a measure of empathy. For this reason, it is suboptimal to combine subscales of the IRI to create a global empathy score: each subscale appears to be relatively independent and unique. More recently, Dermody et al. (37) also used the IRI to examine the neural bases of “cognitive” and affective empathy deficits in Alzheimer's Disease (AD) and bvFTD. While there was a cognitive empathy (i.e., IRI perspective-taking) deficit in both AD and bvFTD, there was an affective empathy deficit only in bvFTD. Deficits in bvFTD, but not AD, remained even after controlling for overall cognitive dysfunction. Perspective-taking deficits in bvFTD were related to bilateral frontoinsular, temporal, parietal, and occipital atrophy, while reduced empathic concern was related to left orbitofrontal, inferior frontal, and insular cortices.

## Future recommendations

Although promising, there are still important caveats to mention about the existing literature on both empathy and perspective-taking. For instance, much of the existing research on these topics uses questionnaire-based measures, such as the IRI (36). While the IRI is extensively used, it is not without methodological concerns. When patients are allowed to self-report, answers are likely biased: Sollberger et al. (38) demonstrated that bvFTD patients overestimate their own empathy on the IRI. Similar results were found by Massimo et al. (39), who showed that patients with bvFTD do poorly when asked to evaluate their own performance on cognitive tests, and by Williamson et al. (40), who asked subjects to predict their performance on tasks of everyday function. Indeed, Eslinger et al. (41) showed that bvFTD patients (termed “social-dysexecutive”) overestimate their performance in 10 of 17 social and emotional domains relative to the judgments of their caregivers. Finally, Rankin et al. (42) demonstrated that, when asked to complete self-report questionnaires about their personalities, patients tend to overestimate their positive qualities and minimize their negative qualities. Patients may also misrepresent their own skills or behaviors for other reasons as well, including apathy. Apathy is frequently documented in patients with bvFTD (43) and could interfere with accurate test-taking abilities. The IRI is also often taken on behalf of patients by their family members or caregivers. Such caregiver and informant-based measures are also problematic. Caregivers are significantly burdened by patient disease, particularly when empathy and/or theory of mind are impaired (44). High levels of caregiver stress may prevent objective ratings and lead to negatively skewed results. Caregiver stress may also vary over the time course of the disease, which would affect the reliability of the data. As mentioned above, the IRI could also be improved by designing more precise subscales. For example, the perspective-taking subscale could be divided into scales for cognitive items and affective items only. Furthermore, in its current form, the empathic concern subscale always probes an individual's tendency to commiserate with another's *suffering*. Empathy, however, is the more general process of sharing another's feelings, whether they are positive or negative. Additional items could be included in the IRI that measure an individual's likelihood to share in another's joy, happiness, or excitement.

In addition to questionnaires, another popular method for assessing empathy and/or perspective-taking includes narrative-based measures. While these methods may be more ecologically valid and do not suffer from the same confounds as questionnaires, narratives and stories are inherently long, which makes them demanding in terms of executive resources. Patients with bvFTD have executive deficits (45, 46), which can potentially confound comprehension. Indeed, some studies have suggested that the impairment of traditional story-based theory of mind tasks may actually be reflective of deficits in working memory, rather than deficits in perspective -taking itself (47, 48).

To address these concerns, future work needs to develop new ecologically valid paradigms that require patients to actively use their perspective-taking abilities. For example, Healey et al. (49) developed a language-based (cognitive) perspective-taking task assessing a patient's sensitivity to the amount of information available to a conversational partner. Unlike narrative-based tasks, resource demands were minimal as patients only had to generate a brief speech sample describing the movement of a target object. Conditions varied depending on perspective-taking demand and how much information was shared with the conversational partner. Results indicated that patients with bvFTD were impaired at this task and that decreased performance was related to gray matter atrophy in medial prefrontal and lateral orbitofrontal cortices. Similarly, instead of using a questionnaire-based metric, Fernandez-Duque et al. (50) used naturalistic stimuli to explore empathy in frontotemporal dementia and Alzheimer's disease. Patients watched videotaped interviews of everyday people discussing emotionally charged events in their lives and answered questions about the interview. This study also demonstrated impaired performance in frontotemporal dementia patients relative to healthy elderly participants. Finally, Baez et al. (51) also highlight the need to use naturalistic stimuli when studying empathy and/or perspective-taking in bvFTD. Baez et al. (51) administered the empathy for pain task (EPT), which uses natural picture stimuli illustrating two individuals in order to assess empathy for another's pain when it is intentional vs. accidental. Following presentation of the picture stimuli, participants were asked to respond to questions in the cognitive domain (e.g., was the action done on purpose?) or affective domain (e.g., how sad do you feel for the victim?) bvFTD patients demonstrated deficits in both the cognitive and affective domains of empathy. The deficit in the cognitive, but not affective, domain could be explained by co-varying for co-existing deficits in executive function, consistent with other reports.

Finally, in examining the differences between cognitive and affective perspective taking, future patients studies need to also examine potential differences in white matter fractional anisotropy. To our knowledge, no study has yet to do this. Similarly, fMRI studies should conduct functional connectivity analyses to see if patterns of network connectivity can differentiate the two types of perspective-taking.

## Conclusions

**Figure 2:**
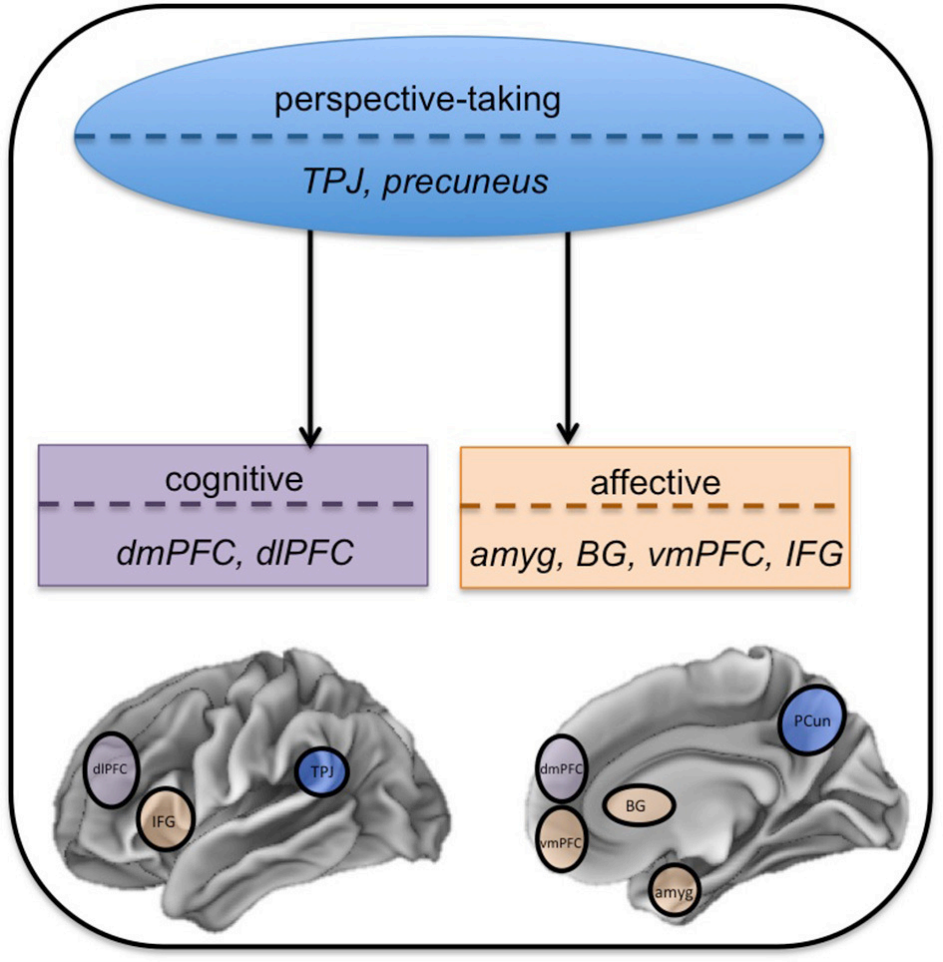
Anatomic model of perspective-taking. In this network approach, the two types of perspective-taking share some cognitive and anatomic substrates. This core perspective-taking module is associated with the temporoparietal junction (TPJ) and precuneus (PCun). Cognitive and affective perspective-taking then diverge into separate components that are functionally dissociable, represented by the two separate boxes. Cognitive perspective-taking, in purple, uniquely engages dorsomedial prefrontal cortex (dmPFC) and dorsolateral prefrontal cortex (dlPFC). Affective perspective-taking, in orange, uniquely engages the amygdala (amyg), basal ganglia (BG), ventromedial prefrontal cortex (vmPFC), and inferior frontal gyrus (IFG).

To date, research seems to suggest that cognitive and affective perspective-taking are in part dissociable. Functional imaging studies have found both shared (e.g., temporoparietal junction, precuneus) and non-shared neural correlates of cognitive and affective perspective-taking, with limbic and basal ganglia structures uniquely involved in affective perspective-taking. There are also regional differences within the frontal lobe between cognitive and affective perspective taking (e.g., cognitive perspective taking elicits activation in dorsal regions, while affective perspective taking elicits activation in more ventral regions). See Figure 2 for a visual depiction of our findings. Perhaps more convincing, however, are the data from patients with behavioral variant frontotemporal degeneration. Behavioral and imaging data in this clinical group show unequal impairment in the two domains, differential relationships with executive function, and unique associations with gray matter atrophy, all of which suggest partially dissociable neural systems. However, there are only a handful of these studies to date, so future research needs to continue to explore empathy and perspective-taking in bvFTD. In doing so, studies must be careful to minimize executive demands, which could confound performance, and try to design stimuli that are as ecologically valid as possible. Finally, across the entire perspective-taking and empathy literature, there is a problem with imprecise terminology (e.g., theory of mind, mentalizing, perspective-taking are all used interchangeably; affective perspective-taking is closely related to cognitive empathy), which makes it difficult to compare and contrast across studies (6). Clear operational definitions must be given whenever possible so we can begin to amass a stronger body of evidence regarding these two constructs.

## Author contributions

All authors listed have made a substantial, direct and intellectual contribution to the work, and approved it for publication.

### Conflict of interest statement

The authors declare that the research was conducted in the absence of any commercial or financial relationships that could be construed as a potential conflict of interest.
